# ANALYSIS OF NOISE GENERATED BY WIND TURBINES WITH REFERENCE TO OTHER LOW FREQUENCY NOISE SOURCES AND THEIR POSSIBLE IMPACT ON HUMAN HEALTH

**DOI:** 10.13075/ijomeh.1896.02433

**Published:** 2025

**Authors:** Andrzej Staniek, Magdalena Miterska

**Affiliations:** Central Mining Institute, Katowice, Poland

**Keywords:** health, annoyance, acoustics, infrasound noise, wind turbines, measurement techniques

## Abstract

**Objectives:**

A problem currently faced in the assessment of human exposure to the external environment concerns sources of noise with significant energy found in the range of infrasound and low sound frequencies. This paper presents an analysis of selected low-frequency noise (LFN) sources in order to demonstrate the problem of the potential exposure of humans residing in their vicinity. There are numerous machines in industry that emit LFN, including infrasound, such as ventilation systems, industrial fans, air and exhaust transfer systems, means of transport and other objects that generate secondary noise, such as acoustic screens. How wind turbine noise differs from noise generated by other sources is a key question.

**Material and Methods:**

There are different measurement approaches concerned with noise monitoring in outdoor environment. For different reasons the measurements are performed at different heights: 4.0 m, 1.5 m, on the ground surface and others. In order to properly identify low frequency noise sources apart from measurement systems for registering sound signals vibration methods might be utilized.

**Results:**

Various types of low-frequency and infrasound noise sources were analyzed in this paper in order to verify the hypothesis concerning the different character of LFN generated by wind turbines. They do not constitute sound sources that generate LFN of higher levels than other sources of this type.

**Conclusions:**

The performed measurements and their analysis revealed that sources of low-frequency and infrasound noise can be found in the vicinity of residential areas, and the residents themselves are unaware of them and consequently do not report the inconvenience related to their emission. Wind turbines are perceived unfavorably by a part of society not only due to their noise, analyzed levels were well below ISO 226 and Møller and Pedersen thresholds, but probably because of other negative aspects such as shadow flicker, modulation or reasons of aesthetics.

## Highlights

- For the investigated objects there is a correlation between acoustic noise measurements and the distribution of vibration signal energy.
- The emission of wind turbine noise is comparable with noise generated by other low-frequency noise sources.
- The issue of noise emissions by wind turbines should not be exaggerated as far as energy production through these sources is concerned.

## INTRODUCTION

### Characteristics of the problem

Global efforts for the improvement of sustainability have led to a rapid increase in the use of renewable energy sources such as wind power [1], which has emerged as a modern source of clean energy relatively recently. The siting of wind turbines has been the subject of an ongoing debate for many years. The reason for this is the potential harmful effect of wind turbines, including hazards caused by infrasound noise. Infrasound means sounds with a frequency <20 Hz. Two types of infrasound sources can be distinguished: natural (unrelated to human activity) and anthropogenic (technical, caused by human activity). Natural sources include wind and all sounds related to it, such as sea waves, waterfalls, storms, volcano eruptions, earthquakes and ocean waves. Technical sources are related to mining activity, explosions and supersonic flight, as well as machine and equipment operation, e.g., compressors, air conditioning and ventilation systems, pumping stations, gas pressure reduction stations, low-speed and high-pressure motors, electric combustion generators, jet and rocket engines, heavy transport vehicles, wind turbines and others [2,3]. Therefore, their occurrence in the natural environment is common. Infrasound is characterized by very high wavelengths, thus it is weakly attenuated and may spread over great distances [2].

### Infrasound generated by wind turbines

Infrasound noise emitted by wind turbines does not reach levels hazardous to humans; it can be compared to the level of natural sound sources common in the environment [3]. Despite that, many individuals claim that the infrasound has a negative influence on their bodies. The topic of infrasound has sparked a polarized public discussion in numerous countries, as some people residing in the vicinity of wind turbines began to report a broad range of health issues and intuitively associated their symptoms with wind turbine-generated infrasound [4]. The perceived annoyance itself varies greatly and depends on the individual. Infrasound imperceptible to one person may be inconvenient to another [5]. Likewise, infrasound tones that are inaudible to some people may be irritating to others [5]. One of the reasons for this are the differences in thresholds of hearing, which may vary even by 20 dB. It was found that individual differences exist and that some people are more vulnerable and susceptible to the influence of infrasound than others [6]. Furthermore, even a slight increase in sound pressure in the low-frequency range may drastically intensify the perceived annoyance [5]. The symptoms reported by people residing in the vicinity of wind turbines include: headaches, nausea, vertigo, tinnitus, ear fullness, arrhythmia, fatigue, sleeplessness, waking up too early, anxiety, stress; illnesses such as hypertension, heart failure, diabetes; as well as the need to take medication for these symptoms, such as painkillers for headaches, joint/muscle pain and other pain as well as medication for sleep disorders, anxiety, depression and hypertension [4].

The most commonly reported symptom resulting from living near wind turbines are sleep disorders. In conducted research, sleep disorders resulting from wind turbine noise were inversely proportional to the distance to the wind turbine [4] and linked to the turbine power; the morbidity of sleep disorders increased together with the increase in the acoustic power level [7]. It is important to differentiate between the notions of noise level and annoyance. There is evidence that annoyance can be significantly higher for the same noise level that includes tonal sounds [8] or amplitude modulation [9]. The reason for this is that annoyance is a psychoacoustic characteristic that describes the spontaneous and undesired reaction to sound, which does not depend exclusively on the objective sound pressure level of the noise source. Persons residing in the vicinity of wind turbines report a greater level of annoyance during early morning, evening and night hours. The annoyance may be caused by high rotational speeds and their variations. During the day, the most complaints were registered for rotations >8 revolutions per min (rpm) [10]. Greater differences between the noise level emitted by the turbines and the acoustic background level are another significant factor.

For turbines with high output power and under the appropriate wind conditions, amplitude modulation interpreted as infrasound can be heard even at a distance of several kilometers and it may intensify the irritation in some individuals [11].

### How infrasound is produced

The noise emitted by a wind turbine is generated by its structural mechanisms as a result of rotor element and generator drive transmission system friction and it is further caused by the air stream produced by the blades [12]. Aerodynamic mechanisms can be named in this context, which encompass the noise of the blade edges and ends, the turbulent flow noise, and impulsive signals resulting from the blade-tower interactions [13,14]. When the blades pass the tower, they encounter variations in the air flow generated by the changes of the wind direction and intensity during its flow around the wind turbine tower. This repeatable process generates impulsive sound signals that consist of a composition of pure tones that are integer multiples of the blade passing harmonics (BPH) – the product of the rotational speed and the number of blades [15–17]. Further-more, the size of the blades and their low rotational speed of 10–30 rpm generates a sound that cannot be placed in the audible frequency range, while a great number of the sounds are emitted as infrasound with a frequency <20 Hz. Van den Berg [17] suggests that it is exactly the blade-tower interaction (BTI) that may be responsible for the wind farm infrasound. In particular, wind turbine blades that move in the air can generate a broad spectrum of sounds, especially low-frequency noise (LFN) within 20–200 Hz, which can spread over great distances, potentially resulting in irritation, sleep disorders and other undesired effects on health [18]. On the other hand, changes to the blade angle of attack result in unstable lift at the blade passing frequency (BPF), which leads to a tonal infrasound and LFN (ILFN, commonly referred to as BTI noise) with characteristic tones at the BPF and higher harmonics [19]. Other possible sources of unstable lift, and thus of ILFN tonality, include cross-winds, atmospheric turbulence and wind shear [19]. Meteorological and acoustic data indicates that the ILFN of a wind farm is likely to be generated under stable atmospheric conditions, which occur the most frequently during night time [19]. Hansen et al. [20] also concluded that wind farm noise is the most perceptible under stable atmospheric conditions and in the direction of the wind.

A tendency for building higher wind turbines with bigger rotors can be observed in the recent decades. They generate more electricity, as the greater height above the ground level provides more stable wind conditions. Modern wind turbines have a hub height of 100 m and more, which is related to the increased rotor diameter and blade end speed. This increase also leads to a higher sound pressure and a greater propagation distance of the aerodynamic noise that can be detected by instruments [21].

## MATERIAL AND METHODS

### Low-frequency noise measurement methodology

Low-frequency noise, including noise generated by wind turbines, consists of the audible acoustic component and infrasound. It is important to take this fact into consideration both when studying human perception and conducting measurements. It is necessary for the frequency range of the measuring apparatus to encompass these areas [21]. The measurement result is always the sum of the noise generated by the analyzed source and the acoustic background.

The general methods of measuring noise levels in the audible range are well known. However, outdoor noise measurements in the infrasound band pose difficulties. Wind-induced microphone noise is a common issue during field infrasound source measurements [22]. The primary problem is the effectiveness of the wind screen at infrasound and low frequencies, which is related to the limitations resulting from its small size [22]. To ensure adequate wind-induced microphone noise reduction and to obtain a satisfactory signal to noise ratio (SNR), wind turbine infrasound assessments require considerably larger wind screens. Furthermore, difficulties in comparing the results exist during wind turbine noise measurements due to the variable conditions (wind speed, proximity of other wind turbines, distance to the wind turbine, etc.) [23]. Currently, the use of ground-positioned microphones and (double) wind screens is a common practice during wind turbine noise measurements [22].

### Infrasound sources in the external environment

To present the influence of noise, including LFN generated by wind turbines, an identification and comparison with other sources in the external environment was performed. Under industrial conditions, infrasound and LFN can be generated mechanically, aerodynamically or hydrodynamically. Its sources can include heavy machinery and equipment, such as transport equipment, as well as metallurgical furnaces, piston compressor systems, ventilation systems, wind power plants, transformers, chimneys, hydraulic networks, closed air and water ducts, pumps, piping systems for gas and process liquid transfer and refrigerating units. Noise sources related to various types of industrial activity and their application were selected for the purpose of analyzing the infrasound noise influence on the external environment. To appropriately identify a source from the perspective of the generated signal frequency range, the surface vibrations of the studied objects were recorded in parallel. A comparison of the industrial infrasound sources with the noise generated by the wind turbines was also performed to answer the question whether these sources differ from those already occurring in the environment, and whether their acoustic annoyance is more significant in terms of their influence on the human body.

### Measuring instruments used

**Figure 1.**
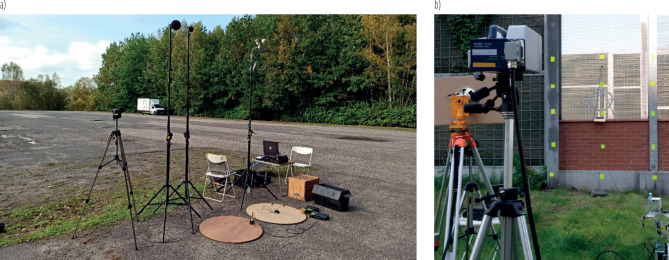
Measurement setups for studies in the field of a) acoustics and b) vibrations, Upper Silesia, Poland, 2024

The measurements were performed using a PSV-400 laser scanning vibrometer (Polytec, Houston, TX, USA) and a PULSE measuring system type 3032 (Brüel & Kjær, Nærum, Dennmark), as presented in Figure 1. The measurement series also utilised 393B12 accelerometers (PCB, Depew, NY, USA) as well as 40AZ (GRAS, Holte, Dennmark) and 4193 (Brüel & Kjær) microphones, shielded by wind screens. Parallel measurements using a SVAN 945A sound level meter (Svantek, Warsaw, Poland) and a 40AN microphone (GRAS) were carried out as well. The measuring equipment fulfilled the requirements of ISO 7196:1995 standard [24]. The measured parameters included: velocity and acceleration (root mean square averaging) for vibration measurements, and equivalent sound pressure level for acoustic noise measurements. The signals were recorded during measurement sessions. It was imperative for the noise source to exhibit stable conditions (normal working parameters), except for the acoustic barriers, where the recordings covered several runs of moving vehicles (separately for passenger car movement and truck movement). For the acoustic noise measurements, a recording time of 10 min was sufficient, though it was subject to several repetitions. For the vibration measurements, the scanning procedure was applied while the recording time depended on the number of points measured. After the measurement phase, a frequency analysis was performed under laboratory conditions. Equivalent sound pressure levels and vibration signal energy distributions in frequency bands were obtained for all the investigated objects.

Photo: Andrzej Staniek

### Studied infrasound sources, acoustic and vibroacoustic methods

The studies encompassed the following objects:

– The central highway (Drogowa Trasa Średnicowa) in the city centre of Katowice at the location of a housing estate characterised by minor distances between the road and the buildings as well as the presence of acoustic barriers.
– Ventilation shafts characterised by the operation of mine fans, the immediate proximity of community gardens and farther proximity of residential buildings.
– A mining plant station in the immediate vicinity of single-family housing infrastructure with the inconvenient noise of diesel locomotives.
– A power unit of gas motors adjacent to a mine and poorly urbanised infrastructure as well as a leisure centre – analysis of a new source of industrial noise.

## RESULTS

### Acoustic barrier

One of the selected objects that constitute sources of LFN are acoustic barriers located in urban areas, particularly in the vicinity of residential buildings. Their basic function is to protect urban and woodland areas from the excess influence of noise, though the movement of vehicles, particularly heavy transport vehicles, induces vibrations on the acoustic barrier surfaces, thus making them a source of LFN. To demonstrate this problem and to identify the LFN source, the results of acoustic noise measurements taken at a distance of about 15 m from an acoustic barrier are collected below (Table 1, noise source A), together with vibration signal energy distribution as measured on the acoustic barrier panel (Table 2, vibration source A). Generally the acoustic signal energy is in a range of ≤250 Hz, with a maximum for the 31.5–63 Hz band, which given the broadband character of the traffic noise confirms the significant influence of these acoustic barriers on secondary emissions in the low-frequency range. The vibration signal energy is dominant in a range of ≤20 Hz. Tables 1 and 2 also contain results for other investigated infrasound sources presented in the following subchapters.

**Table 1.** Equivalent sound pressure levels in individual frequency bands – direct measurements, Upper Silesia, Poland, 2024

Investigated noise source	Equivalent sound pressure level [dB]					
	0–20 Hz	20–31.5 Hz	31.5–63 Hz	63–125 Hz	125–250 Hz	total
A. Acoustic barrier, at a distance of about 15 m from a screen panel						
M1	63.7	67.4	70.3	66.0	55.2	73.8
M2	62.5	66.9	72.0	67.2	54.9	74.9
B. Diesel locomotive, at a distance of 10 m from the source						
M1	79.3	83.3	95.4	87.1	78.7	97.1
M2	76.7	82.9	95.5	87.7	79.2	97.4
C. Gas motor, at a distance of 5 m from a building wall of the source						
M1	85.3	73.8	78.2	90.5	91.0	95.8
M2	102.6	71.2	77.0	92.9	86.9	103.2
D. Ventilation shaft, at a distance of 20 m from the fan enclosure						
M1	90.9	81.6	84.9	87.3	85.2	94.5
M2	82.6	80.1	78.6	82.4	79.2	88.0

M1 – microphone No. 1; M2 – microphone No. 2.

**Table 2.** Percentage contribution of vibration signal energy across frequency bands – direct measurements, Upper Silesia, Poland, 2024

Band	Vibration signal energy [%]			
	A	B	C	D
0–20 Hz	88.3	3.8	4.8	97.9
20–31.5 Hz	8.5	1.0	0.8	1.2
31.5–63 Hz	1.1	9.7	19.1	0.6
63–125 Hz	2.0	11.8	35.8	0.3
125–250 Hz	0.1	65.2	39.5	0.02
250–400 Hz	–	8.5	–	–

In the signal registered on: A – the screen panel of an acoustic barrier; B – the load–bearing frame of a diesel locomotive; C – the gas motor enclosure; D – the fan outlet enclosure.

**Figure 2.**
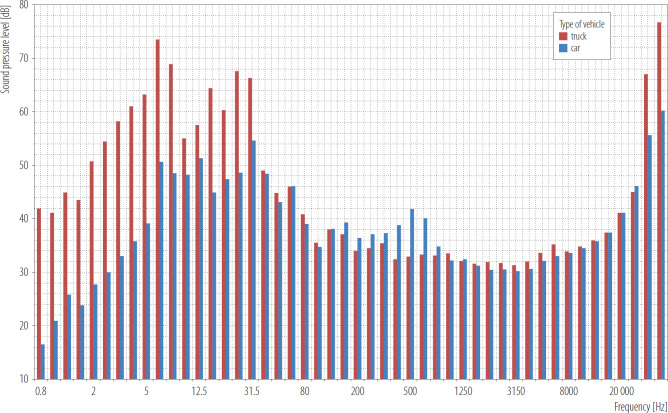
Equivalent sound pressure level in 1/3 octave bands in a living room on the second floor level (passenger car and truck movement), Upper Silesia, Poland, 2024

Acoustic noise measurements were additionally performed in a living room on the second floor level, as presented in Figure 2 and Table 3, which details the movement of passenger cars and trucks. Low-frequency components are visibly dominant in the signal measured for heavy transport.

**Table 3.** Equivalent sound pressure levels in 1/3 octave bands in a living room on the second floor level (passenger car and truck movement) – direct measurements, Upper Silesia, Poland, 2024

Frequency	Equivalent sound pressure level [dB]
Passenger car movement	
0.8 Hz	16.5
1.0 Hz	20.9
1.3 Hz	25.8
1.6 Hz	23.8
2.0 Hz	27.7
2.5 Hz	30.0
3.1 Hz	33.0
4.0 Hz	35.8
5.0 Hz	39.1
6.3 Hz	50.6
8.0 Hz	48.5
10.0 Hz	48.2
12.5 Hz	51.3
16.0 Hz	44.9
20.0 Hz	47.4
25.0 Hz	48.6
31.5 Hz	54.6
40.0 Hz	48.4
50.0 Hz	43.1
63.0 Hz	46.1
80.0 Hz	39.0
100.0 Hz	34.7
125.0 Hz	38.1
160.0 Hz	39.3
200.0 Hz	36.4
250.0 Hz	37.1
315.0 Hz	37.3
400.0 Hz	38.8
500.0 Hz	41.8
630.0 Hz	40.1
800.0 Hz	34.8
1000.0 Hz	32.2
1250.0 Hz	32.4
1600.0 Hz	31.2
2000.0 Hz	30.4
2500.0 Hz	30.5
3150.0 Hz	30.2
4000.0 Hz	30.6
5000.0 Hz	32.1
6300.0 Hz	33.0
8000.0 Hz	33.6
10 000.0 Hz	34.5
12 500.0 Hz	35.8
16 000.0 Hz	37.4
20 000.0 Hz	41.1
Total A	46.1
Total C	55.6
Total Lin	60.2
Truck movement	
0.8 Hz	41.9
1.0 Hz	41.1
1.3 Hz	44.9
1.6 Hz	43.5
2.0 Hz	50.7
2.5 Hz	54.4
3.1 Hz	58.2
4.0 Hz	61.0
5.0 Hz	63.2
6.3 Hz	73.5
8.0 Hz	68.9
10.0 Hz	55.0
12.5 Hz	57.5
16.0 Hz	64.4
20.0 Hz	60.3
25.0 Hz	67.6
31.5 Hz	66.3
40.0 Hz	49.0
50.0 Hz	44.8
63.0 Hz	46.0
80.0 Hz	40.8
100.0 Hz	35.5
125.0 Hz	38.0
160.0 Hz	37.1
200.0 Hz	34.0
250.0 Hz	34.5
315.0 Hz	35.4
400.0 Hz	32.4
500.0 Hz	32.9
630.0 Hz	33.3
800.0 Hz	33.1
1000.0 Hz	33.5
1250.0 Hz	32.1
1600.0 Hz	31.6
2000.0 Hz	31.9
2500.0 Hz	31.7
3150.0 Hz	31.3
4000.0 Hz	32.0
5000.0 Hz	33.6
6300.0 Hz	35.2
8000.0 Hz	33.9
10 000.0 Hz	34.8
12 500.0 Hz	35.9
16 000.0 Hz	37.4
20 000.0 Hz	41.1
Total A	45.0
Total C	67.0
Total Lin	76.7

### Diesel locomotive

**Figure 3.**
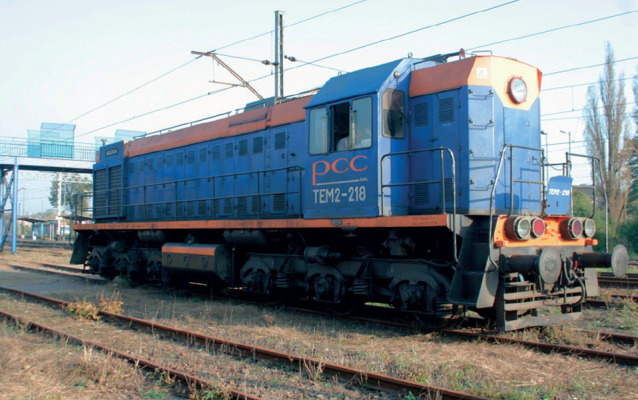
TEM-2 (SM-48) diesel locomotive

The next tests involved the noise emitted by a TEM-2 (SM-48) diesel locomotive awaiting entry or exit from a mine station, presented in Figure 3. This source is responsible for a major part of the acoustic climate in this area and it exhibits a negative influence on the nearby housing infrastructure.

Photo: Andrzej Staniek

The results of acoustic noise measurements taken at a distance of about 10 m from the locomotive are presented in Table 1, noise source B together with vibration signal energy distribution as measured on the diesel locomotive's load-bearing frame, Table 2, vibration source B. Generally, the acoustic signal energy is in a range of ≤250 Hz, with a maximum for the 31.5–63 Hz band. The vibration signal energy is clearly dominant in the 125–250 Hz band.

### Gas motor

The tests also encompassed a power unit whose immediate vicinity includes mining plant infrastructure as well as a housing area and a leisure centre. The conducted measurements and analyses demonstrated that gas motors are a significant source of industrial noise in the case of this power unit. Here, the studies also involved both acoustic noise and mechanical vibration signal measurements. The vibration signal energy distribution in the signal registered on the gas motor enclosure together with the equivalent sound pressure level values in individual frequency bands are presented in Tables 1 and Table 2, accordingly noise and vibration source C. For this source, the predominant sound pressure levels in the noise are found in ranges of ≤20 Hz, and 63–250 Hz, where the vibration energy is also dominant.

### Primary mine ventilation fans

Primary mine ventilation fans constitute a significant stationary source of acoustic noise operating in a continuous manner. The following sources of that noise can be identified in a primary fan unit:

– aerodynamic noise emitted from the diffuser outlet – its acoustic power is proportional to the sixth power of angular speed and squared geometric dimensions of the diffuser;
– fan driving motors located inside the station building, constituting a “building-type” secondary noise source;
– inadequate tightness of reverse flaps and ventilation ducts.

For an example case the measurement results are presented in Tables 1 and Table 2, noise and vibration source D. The vibration signal energy was strongly dominant in the low-frequency range of ≤20 Hz while the sound pressure levels were slightly dominant. Though the acoustic signal is of a more broadband character, the low frequency range is prevalent in the total signal.

It was also found that at a distance of 150 m from the source, a LFN at a sound pressure level of about 70 dB was predominant in the propagating acoustic wave.

### Comparison of the signal spectrum for noise generated by a wind turbine and a ventilation shaft

**Figure 4.**
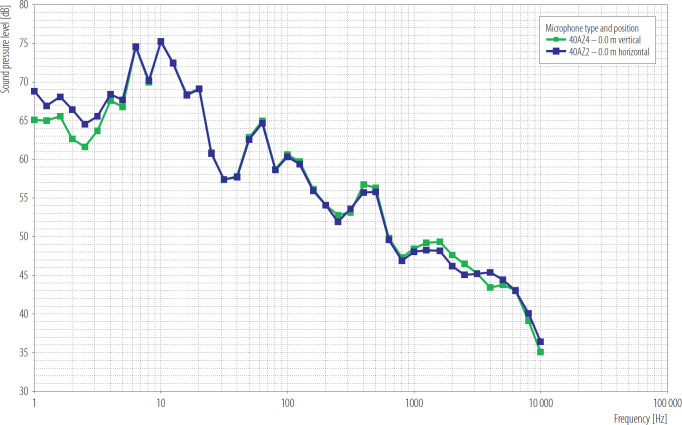
Comparison of the signal spectrum for a microphone mounted in the vertical and horizontal positions corrected, Upper Silesia, Poland, 2024

The placement of the microphone is a significant problem during low-frequency sound measurements. In many cases, infrasound phenomena are the result of air mass movements that provoke significant wind speeds. Wind is an inseparable factor that disrupts measurements in studies concerning wind turbines. Therefore measurement methods utilise microphone mounting on a plate at the ground level [25]. A comparison of the signal spectrum for a microphone mounted in the vertical and horizontal positions is presented in Figure 4. The vertical mounting is suggested in this study, as proposed in the SINTEF project [26] and contrary to the method provided in Standard EN 61400-11 Wind energy generation systems, Part 11: Acoustic noise measurement techniques [27], which proposes horizontal mounting. Vertical mounting ensures better omnidirectionality and a lower influence of wind, especially at low frequencies.

**Figure 5.**
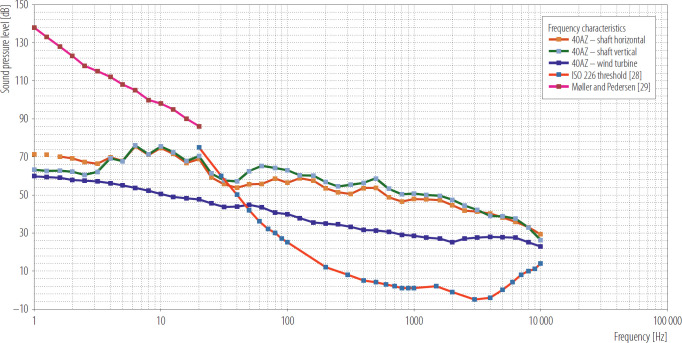
Frequency characteristics for measurements for a ventilation shaft vs. a wind turbine, Upper Silesia and Łódź Voivodeship, Poland, 2024

To illustrate the noise level generated by wind turbines, a compilation of the frequency characteristics for measurements carried out in the vicinity of a ventilation shaft (microphone in 2 positions, vertical and horizontal) and a wind turbine is presented in the Figure 5. Additionally, the characteristics are presented with reference to the threshold levels included in Standard ISO 226 and as proposed by Møller and Pedersen [28,29].

When presenting the results, the measurement uncertainty should also be taken into account. To calculate the expanded uncertainty for the acoustic noise measurements, it was assumed that the measuring equipment was of accuracy grade 1 (calibrated) and that the operating (concerning the noise source) and mounting conditions were stable. Taking that into consideration, the expanded uncertainty for the sound emission of the investigated object may be approximated at 1.4 dB [30].

According to laboratory procedures, the expanded uncertainty for the vibration measurements may be approximated at 16% after factoring in vibration transducers and the measurement unit.

## DISCUSSION

### Human reaction

It is commonly acknowledged that social aspects play an important role in the introduction of wind power, and social participation in decision-making contributes to its acceptance by citizens. Wind turbines are perceived unfavourably by a part of society not only due to their noise, but also because of their potentially negative influence on the human body. It is justified to state that the annoyance caused by wind turbines is related to the character of their generated signal (modulation) and the visibility of the structures themselves. Results indicate that the most important factors considered during the acceptance of wind power projects include the noise level in the area of residence, the distance from the turbines to the area of residence, and the possibility for citizens to take part in the project planning through information, consultation, cooperation and financial participation [31].

Respondents who obtained economic benefits from agreeing to the installation of wind turbines reported significantly lower irritation [32]. Furthermore, in another study, despite experiencing more frequent and increased noise levels, the respondents who obtained economic benefits reported considerably lower irritation relative to those respondents who did not obtain such benefits [33].

One of the key aspects in siting wind turbines is the reduction in the value (land price) of local real estate, where the primary parameters include the proximity and/or visibility of the wind turbine [30]. Among the persons irritated by the sound, about two-thirds of the respondents did not appreciate the appearance of the wind turbine, which suggests that a certain relationship may exist between visual and auditory reactions and the influence of a wind turbine [34]. In the conducted researches, it was demonstrated that the problem of infrasound emissions is much broader and that it should not be limited solely to the noise generated by wind turbines [34,35]. At the same time, the issue of noise emissions by wind turbines should not be exaggerated as far as the production of energy by means of these sources is concerned. They do not constitute sound sources that generate LFN of higher levels than other sources of this type.

## CONCLUSIONS

An analysis of selected LFN sources was performed in order to demonstrate the problem of the potential exposure of humans residing in their vicinity. In industry, ventilation systems, including industrial fans and other equipment used in air and exhaust transfer systems, may emit noise for which a major part of the energy in the spectrum is found in the infrasound and low sound frequency ranges. In further studies, it could be valuable to introduce G frequency weighting for the measured signals [36,37] to assess their impact on human health (as recommended in standard ISO 7196:1995), though the aim of this research was to compare different sources of infrasound and low frequency noise.
